# Editorial: The role of different physical exercise protocols on immunological and immunometabolic profile in physiological and chronic diseases

**DOI:** 10.3389/fimmu.2023.1327967

**Published:** 2023-11-09

**Authors:** Fabrício Oliveira Souto, Angela Castoldi, Eduardo Zapaterra Campos, Tomasz Kostka

**Affiliations:** ^1^ Keizo Asami Institute (iLIKA), Federal University of Pernambuco (UFPE), Recife, Brazil; ^2^ Graduate Program in Physical Education, Sports Performance Research Nucleus (NIDE), Federal University of Pernambuco, Recife, Brazil; ^3^ Department of Geriatrics, Healthy Ageing Research Centre, Medical University of Lodz, Lodz, Poland

**Keywords:** physical exercise protocols, chronic diseases, immune system, immunometabolism, physiology

Physical exercise has long been recognized for its numerous health benefits, including cardiovascular and mental well-being (1). However, recent research has shed light on the intricate relationship between physical exercise and the immune system (2). In this Research Topic, “*The Role of Different Physical Exercise Protocols on Immunological and Immunometabolic Profile in Physiological and Chronic Diseases*,” six manuscripts were accepted for publication, covering a wide range of research, including three original research articles, one clinical trial, one mini-review, and one comprehensive review.

The article by Costanti-Nascimento et al. explores the therapeutic potential of regular and moderate exercise in acute kidney injury (AKI). While extenuating physical exercise has been associated with AKI due to its severe impact on the body’s physiology, this study uncovers a different perspective. In AKI induced by various factors, including Ischemia/Reperfusion, Sepsis, Diabetes, Antibiotics, and Chemotherapy, regular and moderated exercise is shown to modulate the immune system towards a regulatory and anti-inflammatory profile. It reduces oxidative stress, inflammatory markers, and cytokines, creating a conducive environment for AKI recovery. This research highlights the importance of considering moderate exercise as a complementary therapy for AKI patients.

The intricate interplay between sedentary behavior, physical exercise, and sleep disturbance was investigated in a manuscript by You et al. This population-based study involving 22,599 participants from the National Health and Nutrition Examination Survey reveals that sedentary behavior is a risk factor for sleep disturbance. However, exercise, especially in severe sedentary behavior groups, can mitigate sleep disturbance symptoms. Importantly, the study elucidates the mediating role of inflammatory biomarkers, such as white blood-cell count and neutrophil-to-lymphocyte ratio, in the relationship between sedentary behavior and sleep disturbance. These findings underscore the importance of physical activity in improving sleep quality and its potential to modulate the immune system.

The impact of supervised and home-based exercise protocols on recovered COVID-19 patients is explored by Filgueira et al. The study demonstrates that supervised exercise can modulate the immune response in post-COVID-19 individuals, suggesting its potential to mitigate the inflammatory processes associated with the disease. The findings underline the importance of exercise as a complementary strategy in the management of COVID-19-related complications.

As the world grapples with the ongoing COVID-19 pandemic, the article by Teo and Goodwill explores the potential of exercise in managing long COVID syndrome (LCS). LCS presents ongoing neuropsychological symptoms, and evidence suggests that altered inflammatory pathways contribute to these symptoms. Exercise has been shown to have positive effects on brain health, including inflammation modulation. The authors argue that exercise could serve as an essential lifestyle factor in managing LCS, emphasizing the need for further research in this area.

In a study on male habitual marathon runners, Panagoulias et al. explore the long-term effects of strenuous exercise on the immune system. Marathon running is known for its intense physical demands, and this research reveals permanent changes in certain immune parameters in marathoners compared to sedentary individuals. Marathoners exhibit a pro-inflammatory cytokine polarization that increases after races, counterbalanced by increased numbers of regulatory T cells (Tregs) with enhanced suppressive capacity. These findings shed light on the important relationship between extreme exercise and immune system adaptations.

Pinto et al. delve into the intricate relationship between interleukin-6 (IL-6) and exercise-induced adaptations in skeletal muscle. IL-6 has a complex role as both a pro and anti-inflammatory cytokine, and this study reveals its involvement in modulating autophagy and mitochondrial metabolism. The downregulation of REV-ERBα, autophagic flux, and mitochondrial genes in IL-6 knockout mice suggests that IL-6 plays a crucial role in exercise-induced adaptations. These findings open new avenues for understanding the molecular mechanisms behind exercise-induced changes in skeletal muscle.

In conclusion, the studies presented in this Research Topic underscore the importance of physical exercise as a modulator of the immune system in various physiological and pathological contexts (3, 4). We are excited to share the valuable insights from these six accepted manuscripts, which provide a comprehensive view of the complex relationship between exercise and the immune system. As we navigate the challenges of the modern world, where diseases and health conditions continue to pose threats, it is clear that different types of physical exercise remain a powerful tool in promoting the body’s physical and immunological health (5). We hope that this Research Topic inspires further exploration of the immune-modulatory effects of exercise and its potential applications in the prevention and management of various diseases.

## Author contributions

FS: Conceptualization, Supervision, Writing – original draft, Writing – review & editing. AC: Conceptualization, Writing – review & editing. EC: Conceptualization, Writing – review & editing. TK: Conceptualization, Writing – original draft, Writing – review & editing.
